# Supplementary material for: Are European clinical trial funders policies on clinical trial registration and reporting improving? A cross-sectional study
Source: J Clin Transl Sci. 2023 Jul 14;7(1):e166. doi: 10.1017/cts.2023.590 (PMC10425870; doi:10.1017/cts.2023.590)
Supplement: Supplementary file 1 [file S2059866123005903sup001.docx]

**Supplemental Content**

Marguerite O’Riordan, Martin Haslberger, Carolina Cruz, Tarik Suljic, Martin Ringsten, Till Bruckner

Are European Clinical Trial Funders Policies on Clinical Trial Registration and Reporting Improving? – A Cross-Sectional Study

**eFigure**: Funder ratings and partial policies

This supplemental material has been provided by the authors to give readers additional information about their work.

**eFigure**. Rating Changes and Partial Policies

The following table provides an overview of:

(a) of non-comprehensive and non-binding funding policies, which did not receive any points in the scoring system used by the study

(b) changes made and declined by the study team based on feedback received from sponsors

S = funder supports/encourages practice without mandating it

P = funder policy item only applies to a specific sub-set of trials (but not to all trials)

BLUE HIGHLIGHTING = change made in response to funder feedback

GREY HIGHLIGHTING = funder requested change but original score maintained
